# Functions of Ionic Liquids in Preparing Membranes for Liquid Separations: A Review

**DOI:** 10.3390/membranes10120395

**Published:** 2020-12-05

**Authors:** Dayuan Zheng, Dan Hua, Yiping Hong, Abdul-Rauf Ibrahim, Ayan Yao, Junyang Pan, Guowu Zhan

**Affiliations:** 1 Integrated Nanocatalysts Institute (INCI), College of Chemical Engineering, Huaqiao University, 668 Jimei Avenue, Xiamen 361021, Fujian, China; zhengdayuan@stu.hqu.edu.cn (D.Z.); 1626211007@stu.hqu.edu.cn (Y.H.); ayan8360@stu.hqu.edu.cn (A.Y.); 20014087024@stu.hqu.edu.cn (J.P.); 2 Department of Mechanical Engineering, Faculty of Engineering and Built Environment, Tamale Technical University, Education Ridge Avenue, Sagnarigu District, Tamale, Ghana; ghrauf@gmail.com

**Keywords:** ionic liquids (ILs), membranes, liquid separation, modifier, solvent

## Abstract

Membranes are widely used for liquid separations such as removing solute components from solvents or liquid/liquid separations. Due to negligible vapor pressure, adjustable physical properties, and thermal stability, the application of ionic liquids (ILs) has been extended to fabricating a myriad of membranes for liquid separations. A comprehensive overview of the recent developments in ILs in fabricating membranes for liquid separations is highlighted in this review article. Four major functions of ILs are discussed in detail, including their usage as (i) raw membrane materials, (ii) physical additives, (iii) chemical modifiers, and (iv) solvents. Meanwhile, the applications of IL assisted membranes are discussed, highlighting the issues, challenges, and future perspectives of these IL assisted membranes in liquid separations.

## 1. Introduction

Membrane-based liquid separation technologies mainly include microfiltration (MF), ultrafiltration (UF), nanofiltration (NF), organic solvent nanofiltration (OSN), reverse osmosis (RO), forward osmosis (FO), and pervaporation (PV). These separation technologies play important roles in the industry and our daily life, because of their functions in the concentration, fractionation, and purification of liquid mixtures. Moreover, the separation technologies have experienced rapid growth in the past decades since they are believed to have fewer energy consumptions, smaller carbon footprints, and convenient operations compared to traditional separation technologies such as distillation, condensation, and crystallization [1]. 

**Figure 1:**
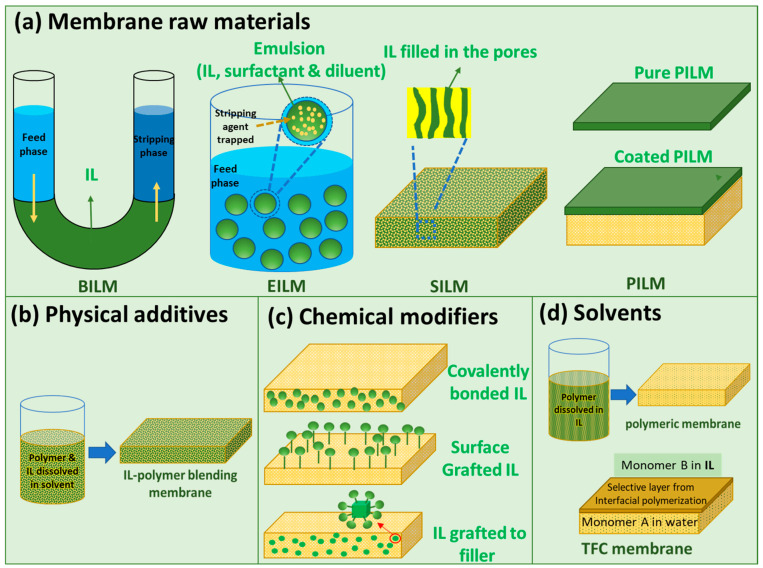
The four major functions of ionic liquids (ILs) in preparing membranes: (**a**) membranes raw materials (BILM: bulk IL membrane, EILM: emulsion IL membrane, SILM: supported IL membrane, PILM: poly (ionic liquid) membrane), (**b**) physical additives, (**c**) chemical modifiers, (**d**) solvents (TFC: thin-film composite).

However, most of the membranes are fabricated through the phase inversion method with the use of toxic organic solvents or strong acids, which often generate toxic volatile organic compounds and produce a large amount of wastewater containing toxic solvents [2]. To circumvent these environmental problems, one direct strategy is replacing the common toxic solvents with greener solvents that have lower volatility or less toxicity, such as TamiSolve^®^ NxG [3], PolarClean [4], Cyrene™ [5], dimethyl isosorbide [6], dimethyl carbonate [7], etc. Ionic liquids (ILs) are also viewed as an alternative green solvent. Specifically, room-temperature ILs are molten organic salts that are in a liquid state at or near room temperatures. They have received tremendous attention due to their excellent properties such as strong polarity, negligible vapor pressure (above the liquid surface), low volatility, thermal and chemical stability, designable structure, and a good ability to dissolve many inorganic, organic, and polymeric materials. Consequently, they have been used in the preparation of various membranes [8,9,10,11,12,13,14,15,16,17,18,19]. The usage of ILs in fabricating membranes could be broadly categorized into four types as illustrated in Figure 1.

**Figure 2:**
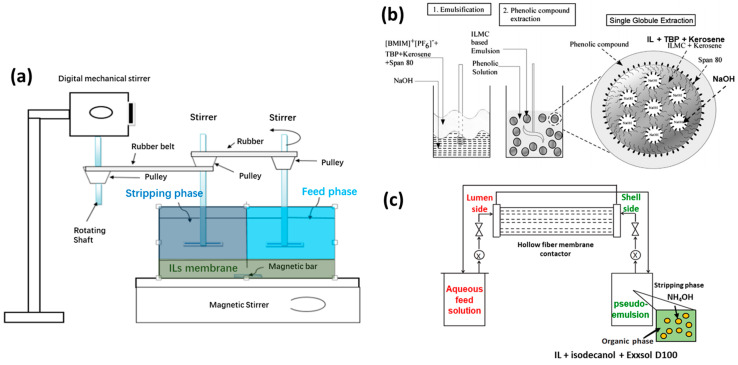
Schematic for (**a**) an extraction unit of 4-nitrophenol compounds using a BILM, adapted and modified from [37]; (**b**) the removal of phenolic compounds using an IL mixed carrier-based EILM, adapted from [46]; (**c**) the pseudo-emulsion based hollow fiber membrane with strip dispersion system, adapted from [48].

ILs can be used directly as raw materials to fabricate membranes. As shown in Figure 1a, some of such membranes include bulk IL membranes (BILMs), emulsion IL membranes (EILMs), supported IL membranes (SILMs), and poly(ionic liquid) membranes (PILMs): (i) BILMs, the simplest non-supported IL membranes, usually consist of the aqueous feed phase and the stripping phase directly contacted with an IL membrane in a U-tube (refer to a setup in Figure 2a); (ii) EILMs are generally emulsions prepared by emulsifying an organic phase (i.e., the carrier containing IL, surfactant, and diluents) with an internal aqueous stripping agent. In the case of EILMs, the emulsion droplets are often stabilized by surfactant, enabling them to trap internal stripping agents inside them and form water-in-oil emulsion [20]; (iii) SILMs are a type of liquid membrane, wherein the IL is held by capillary forces in the pores of a polymeric/inorganic membrane support via direct immersion, vacuum infiltration, etc. The ILs play an important role in the operating performance of SILM due to the relatively high viscosity; (iv) PILMs are directly made of poly(ionic liquid)s (PILs), which are polyelectrolytes that feature IL species in each monomer repeating unit and connected through a polymeric backbone to form a macromolecular architecture. The synthesis methods for PILs could be referred to in a report developed by Yan et al. [21]. The PILMs could normally be made from PILs solutions via a phase inversion process and coating on a membrane support. As compared to the liquid membranes, PILMs made from high molecular weight PILs are much more stable, which form adjustable structures and morphologies [21].

The first three types of membranes (BILMs, EILMs, and SILMs) are types of solvent extraction based on liquid membranes. The IL membranes are much greener and more stable as compared with traditional liquid membranes made from organic solvents, meaning that the usage of ILs overcomes the evaporation loss of organic solvents. Among these types of membranes, BILMs have the lowest contact surface area for extraction while EILMs have the largest contact surface area. Hence, the permeation rate of BILMs is very low, making them technologically not very attractive. However, BILMs are the simplest membranes and are still widely used to study the transport properties of novel ILs as carriers [22]. EILMs have the advantages of a high surface area, non-dependence on equilibrium consideration, and relatively low cost, but their stability is a critical issue because the emulsions formed should be stable enough to avoid leakage during the separation process. On the other hand, the emulsions should not be too stable so that they can be destroyed and recycled after the separation process. Although the SILMs have lower flux as compared to EILMs due to having less contact surface area, high selectivity could be achieved based on small amounts of ILs. Thus, SILMs have gained much popularity in recent years. Similarly, ILs and polymerized ILs (PILs) have been used as physical additives into membranes to form IL/PIL-polymer blending membranes (Figure 1b). The IL-polymer blending membranes are also called polymer inclusion membranes (PIMs). As compared with pristine IL membranes, they possess improved stability due to the fact that ILs are trapped in the dense polymeric/inorganic membrane matrix. As compared with pristine membranes, they may have enhanced separation performance due to the special physical properties of ILs such as hydrophilicity, charge, special functional groups, etc. Moreover, ILs/PILs have been used to chemically modify the membranes or membrane components owing to the abundant active functional groups. As illustrated in Figure 1c, on one hand, ILs/PILs could be added into polymer bulk solutions and form a membrane layer possessing excellent stability due to covalent bonds; on the other hand, ILs could also be chemically grafted to the membrane surface or serve as membrane additives to improve the separation performance. Furthermore, ILs have been used as green solvents for dissolving polymers or as reaction media during membrane fabrication (Figure 1d). Due to the electrostatic nature of ILs, they are able to interact strongly with the polymers via pronounced hydrogen bonding, Coulombic forces, and van der Waals interactions [23,24,25]. Using IL as the solvent is greener than using organic solvents because ILs are non-volatile and can be recovered in some cases. Moreover, they can be used to dissolve some rigid polymers (e.g., cellulose, polybenzimidazole (PBI), and polyamides) which are not easily dissolved by organic solvents [25,26,27]. Besides, using ILs as the reaction media benefits not only from their non-volatility, but also from their different properties such as interfacial tension, viscosity, and the solubility of organic compounds, making the membrane synthesis less hazardous and more tunable to obtain membranes with better separation performance [28].

Therefore, ILs play an important role in membrane fabrication from the versatile aspects shown above. Currently, there have been many studies on developing these types of IL assisted membranes for gas separations (such as CO_2_ and volatile organic compounds removal) because of their high solubilities for different gaseous species [10,11,15,17]. Besides this, the use of ILs has also been extended to fabricating membranes for other purposes such as electrochemical applications [29,30,31,32,33], osmotic power generation [16], liquid separations including desalination, the removal of organics from water or organic solvents [34,35], removal of heavy metal [36], organic solvent/water separations, and so forth. Although the ILs have been used extensively in developing membranes for liquid separations, few reviews have summarized the functions of ILs in the rational design of these membranes. Besides, the existing reviews are limited to IL membranes, in which ILs are used as fabricating materials [12,18,19]. 

Considering that liquid separations are in fact, more prominent than gas separations in industrial membrane separation processes [1] and that ILs have more versatile applications in membrane fabrications, it is thus important to systematically summarize the functions of ILs in developing membranes for liquid separations. In this regard, this review provides a comprehensive overview of four major functions of ILs in developing liquid separations membranes. Moreover, problems and challenges in IL related membrane processes for liquid separations are identified and discussed.

## 2. Functions of ILs in Developing Liquid Separation Membranes

### 2.1. ILs as Raw Membrane Materials

ILs have very low vapor pressure but they have good solubility for organic and inorganic compounds. Therefore, they can be used as materials directly to fabricate membranes such as bulk IL membrane, supported IL membrane, emulsion IL membrane, and poly (IL) membrane. Recent progress in the development of each of these membranes will be summarized in the following sections.

#### 2.1.1. Bulk IL Membrane (BILM)

Unlike conventional bulk liquid membranes, BILMs use ILs as the hydrophobic liquid membrane phase instead of organic solvents, which has attracted the interest of researchers due to the ‘green properties’ of ILs such as low vapor pressure, low volatility, and good stability at high temperatures, making the BILM more stable and less hazardous because of the reduced evaporation of the membrane phase.

Several studies on the use of BILMs to remove various organic compounds in the liquid solutions have been reported, including phenols [37,38], organic acids [39,40,41], and others [39,40,42,43,44]. Lakshmi et al. utilized three different highly hydrophobic ILs to study the removal efficiency for chlorophenol [38]. Interestingly, high chlorophenol extraction and stripping efficiencies of 98.10% and 78.5%, respectively, were achieved by using 1-butyl-3-methylimidazolium tetrafluoroborate ([BMIM][BF_4_]) with minimum membrane loss. Similarly, Mohammed and Hameed synthesized several hydrophobic ILs for extracting 4-nitrophenol from an aqueous solution [37]. They found that the distribution coefficients for the 4-nitrophenol in the ILs were higher than in conventional organic solvents. Furthermore, 1-butyl-3-methylimidazolium bis(trifluoromethylsulfonyl)imide ([BMIM][Tf_2_N]) exhibited the greatest extraction and stripping efficiencies. Baylan and Çehreli used four hydrophobic imidazolium-based ILs as the membrane, tributyl phosphate (TBP) as a carrier for the membrane, and NaOH solutions as the stripping phase to remove levulinic acid [41] and acetic acid [40] from the aqueous solutions. Their results indicated that all the investigated ILs as a membrane phase had good transport selectivity. Moreover, TBP and NaOH concentrations had a major influence on both the extraction and stripping efficiencies. In addition, Branco et al. performed a systematical selective transport study using a 7-component mixture of representative organic compounds, and 10 different ILs based on five cation structures. Remarkably, they observed that the use of ILs with more polar cations (containing ether or hydroxyl functional groups) generally increased their affinity for all organic compounds but reduced the selective transport, especially for secondary and tertiary amines. Chakraborty and Bart successfully used 1-octyl-3-methylimidazolium chloride as a membrane solvent and Ag^+^ as the carrier to remove toluene from n-heptane [42]. They revealed that the Ag^+^ concentration, stirring speed, initial toluene concentration in the feed, and temperature greatly influenced the permeation rate and separation factor. 

There have also been a few studies on the use of BILMs to remove metal ions from the aqueous solutions. For instance, Kogelnig et al. conducted a successful quantitative transport of Fe(III) ion from a hydrochloride (6 M) feed phase containing Ni(II) to a hydrochloride (0.5 M) receiving phase by using a commercialized IL trihexyl(tetradecyl)phosphonium chloride (Cyphos^®^ IL101) as the membrane phase [45]. Both of the two metal ions have an ion association with the chloride anion to form a complex. The separation mechanism was based on the difference in the complex behavior depending on the concentration of HCl.

As can be seen from above, the studies on the use of BILM are very limited, especially the applications on metal ion removal. For instance, most researchers have screened several potential hydrophobic ILs to develop a suitable BILM for a certain organic solute. In addition, the selections of a suitable carrier and stripping agent have been found to be critical. Also, other operation factors (such as the feed phase, feed pH, carrier concentration, stripping agent concentration, contact time, stirring speed, temperature, etc.) greatly influence the permeation rate and separation factor. By tuning those parameters, the separation performance could be further enhanced, but BILMs are technologically unattractive due to their low contact surface area and slow mass transfer rate [22]. Despite this, BILMs are still meaningful because they are normally employed to study the transport properties of the novel carriers [22,43], which could give guidance for further developing other types of IL membranes.

#### 2.1.2. Emulsion IL Membrane (EILM)

EILMs have a much higher surface area per unit of volume and lower thickness as compared to BILM because the membrane phase is made of numerous small emulsion droplets containing ILs, making the separation process and accumulation inside the emulsion vehicle fast. Similarly, the usage of ILs in the membrane emulsion makes the liquid membrane system more stable, which has been proved by the following studies.

Balasubramanian and Venkatesan built an EILM system by using a mixture of 1-butyl 3-methylimidazolium hexafluorophosphate and TBP as a mixed carrier, Span 80 as a surfactant, kerosene as a diluent agent, and NaOH as the internal stripping agent [46]. The EILM system was then applied for the removal of phenolic compounds; the scheme is illustrated in Figure 2b. They optimized the system parameters for achieving maximum removal of phenol with a higher treat ratio. The various parameters include the concentrations of IL, TBP, stripping reagent, surfactant, emulsification time, phase volume ratio, treat ratio, stirring speed, and external phase pH. Even though the IL was not purely the carrier in the system, the addition of the IL in the membrane phase increased the stability of the emulsion over 5 folds than that of the emulsion without the IL. Moreover, Kulkarni’s group established an EILM system by using di-2-ethylhexyl phosphoric acid and 1-octyl-3-methylimidazolium hexafluorophosphate ([OMIM][PF_6_]) as a carrier, Span 80 as a surfactant, hexane as a diluent agent, and sulphuric acid as an internal stripping agent [47], which was then used to remove Pb (II) ions from aqueous solution. Similarly, the various operating parameters were investigated and optimized. They found that the stability and the enrichment factor of the EILM were 2-3 folds greater than those for the system without the IL.

In addition, Alguacil et al. reported a smart IL membrane technology based on microporous polypropylene hollow fiber membrane contactor for the removal of Cd (II) from acidic chloride solutions. In this case, the Cd (II) feed solution and the pseudo-emulsion of the organic solution (tri-isooactylammonium chloride IL + isodecanol + Exxsol D100) plus the stripping agent (NH_4_OH) were not directly in contact. Actually, they passed through the lumen side and shell side of a membrane contactor in a counter-current mode, as shown in Figure 2c [48]. After the optimization of several different parameters, efficient removal of Cd(II) with a mass transfer coefficient value of $2.68\times 10^{-4}$ cm/s was achieved. The authors believed their methodology was a promising alternative to conventional procedures because it combined the operational characteristics of liquid membranes and liquid-liquid extraction technologies. However, further studies on comparing this system with the traditional ones are needed to confirm the removal efficiency.

To sum up, the stability of EILMs is greatly enhanced as compared to the traditional emulsion liquid membranes with only organic solvents as the carrier, but the swelling and breakage of emulsion still exist during the separation process, which could be alleviated by optimizing the operational conditions (such as IL concentration, surfactant concentration, agitation speed, extractant concentration, etc.). Besides, other types of surfactants and ILs that have better chemical interactions could be investigated, since that they can avoid the coalescence of internal phase droplets and enhance stability [47].

#### 2.1.3. Supported IL Membrane (SILM)

Similarly to EILMs, SILMs are more stable than a traditional supported liquid membrane owing to the negligible evaporation of IL. Compared with BILM and EILM, the SILM requires much less membrane solvent and yet, offers higher selectivity [22] making it more popular for membrane fabrication. There have been several review papers reporting the research progress of SILMs and their applications in the separation of organic compounds, gases, vapors, ions, and so on [12,22,49,50,51,52]. In this review, we focus more on SILM related research works for liquid separations reported in the last 5 years. PV is a membrane process where the liquid feed is in direct contact with one side of the membrane, while the permeate evaporates into sweeping gas or vacuum on the other side. Generally, the applications of PV include (i) dehydration of organic-water mixtures, (ii) removal of trace organic compounds from water, and (iii) organic-organic mixture separation. Currently, developed SILMs for PV are mainly applied in the latter two applications, with hydrophobic ILs as the membrane phase. According to the reports that were published previously, recovery of butanol from aqueous mixtures is the most investigated way to remove trace organic compounds from water [49,51]. In recent years, SILMs have been explored for separating other mixed systems, especially those organic-organic mixtures.

Mai et al. fabricated a SILM by depositing 1-octyl-3-methylimidazolium bis(trifluoromethylsulfonyl)imide ([OMIM][Tf_2_N]) on a polydimethylsiloxane (PDMS) support in vacuum. The SILM was then used to recover acetone, butanol, and ethanol (ABE) from an aqueous solution by using PV [53]. However, they found that the SILM had a lower permeation flux and lower selectivity compared to the immobilized IL-PDMS membrane. Zhang et al. developed a SILM system for separating toluene and cyclohexane by impregnating porous polyvinylidene fluoride (PVDF) hollow fiber membrane with ILs [54]. They studied the interactions of several ILs with toluene and cyclohexane by using quantum chemical calculations and the liquid-liquid extraction process. The results showed that N-Butylpyridinium tetrafluoroborate ([BPy][BF_4_]) has a stronger interaction with toluene than cyclohexane, and it also showed good long-term stability of over 100 h due to its good affinity for the hollow fiber support and the high viscosity. Luis’s group prepared two SILMs based on [OMIM][Tf_2_N] and N-octyl-N-methylpyrrolidinium bis(triuoromethanesulfonyl) imide by using a vacuum for PV separation of dimethyl carbonate (DMC)/methanol mixtures [55]. They found that the separation factor (methanol relative to DMC) of the SILMs was highly dependent on the feed concentration, which was high only at a high DMC concentration of 0.8 M. 

Meanwhile, SILMs have also been used to remove organic compounds from aqueous solutions based on extraction. Fortunato et al. fabricated an [OMIM][PF_6_] based SILM to extract amino acids or amino acid esters [39]. They found that the IL had a better selectivity for amino acid esters. However, the SILMs showed a significant loss of selectivity in a short testing period (2-4 h). This according to them could be ascribed to two reasons: (1) the loss of the organic phase from the membrane support to the adjacent aqueous solution caused by dissolution/emulsification; (2) the formation of water microenvironments inside the organic phase, which constitute new and non-selective environments for solute transport. Matsumoto et al. prepared several SILMs for separating lactic acid by impregnating 6 commercial ILs into the pores of the PVDF MF membrane support using the direct immersion method [56]. They found that CYPHOS IL-104 SILM showed a very low permeation rate, whereas the CYPHOS IL-109 and -111 SILMs were unstable due to the loss of IL from the membrane support. Aliquat 336, CYPHOS IL-101, and CYPHOS IL-102 were found to be suitable in terms of the membrane stability and permeation of lactic acid. The same ILs and hydrophilic PVDF were also used to fabricate SILMs to remove phenol from aqueous solutions by Pilli et al. [57]. For this case, CYPHOS IL-104 gave the highest permeation rate. However, it also showed a quick decline of permeation in the first 10 h although the decline was much less in the later evaluation time. Nevertheless, a longer testing time is still needed to further confirm the stability of the membranes in this study. Panigrahi et al. also fabricated several SILMs using PVDF as the membrane support by a direct immersion method, and the SILMs were then used to separate Bisphenol A (BPA) from the aqueous solution [58]. They got a BPA permeation rate order among different ILs and claimed that the IL weight loss was less than 2% after 24 h. However, their study was a preliminary one and the maximum permeation of BPA they reported needed to be improved upon. Abejón et al. studied five different membrane supports and nine ILs for removing or selective transport of two different technical lignins (i.e., Kraft lignin and lignosulphonate) and monosaccharides (xylose and glucose) in an aqueous solution [59]. However, only the SILM composed of 1-butyl-3-methylimidazolium dibutylphosphate and polytetrafluoroethylene membrane support allowed for the selective transport of the tested solutes. Some of their ILs dissolved some of the membrane supports, whereas others experienced precipitation. Moreover, the stability and separation efficiencies of their SILM need further studies.

Similarly, SILMs have also been applied for metal removal. Jean et al. reported the extraction of Hg(II), Cd(II), and Cr(III) ions from acidic media with a SILM using a novel synthesized IL (isooctylmethylimidazolium bis-2-ethylhexylphosphate) as the carrier [60]. The SILM was more suitable for the extraction of Cd (II) ions. During stability investigation, 11% of the IL was released after 4 cycles. Zante et al. evaluated the feasibility of selectively separating lithium cations from aqueous solutions containing sodium, cobalt, and nickel ions using a SILM fabricated by impregnating porous PVDF membrane support with a mixture of hydrophobic IL [BMIM] [Tf_2_N] and TBP as the carrier [61]. Very importantly, their stability experiments indicated that the loss of IL into the aqueous phase could be reduced by the addition of salt in the feed or the stripping phase. 

Although the SILMs have been widely studied for various liquid separations, stability issues a concern in some cases, which is due to the gradual solubilization or emulsification of the liquid phase of the membrane (carrier or organic solvent) in the surrounding aqueous phase. As indicated by the literature above, enhancing the interactions between the IL and membrane support is critical. Accordingly, various strategies to improve the stability are considered when developing SILMs, such as screening the strong affinity between the membrane support and IL, chemical modification, minimizing the pores of membrane supports to nano-size level, or directly mixing IL and another polymer prior to membrane casting or impregnation in the pores of membrane supports.

#### 2.1.4. Polymerized IL Membrane (PILM)

As a type of polymer, PILs are more suitable to be directly used as membrane materials than ILs because they possess both the designability of ILs and the selectivity of polymer segments. The use of PILMs in separations offer undoubtedly engineering and economic advantages over other separation technologies for CO_2_ capture from fossil fuels and flue gas streams, as well as in CH_4_ separation and purification [21,62]. Moreover, PILs have already been studied as novel polyelectrolyte membranes and electrolytes for batteries, fuel cells, and dye-sensitized solar cells [62,63]. Considering that PILs could interact with other molecules through hydrophobic and hydrophilic interactions, hydrogen bonding, ion exchange, π-π stacking, or electrostatic interactions, PILMs have also been extended to several liquid separations, including the removal of metals, dyes, desalination, the concentration of proteins, oil/water separations, etc. To design these PILMs membranes with different structures for different applications, various fabrication methods have been developed. 

**Figure 3:**
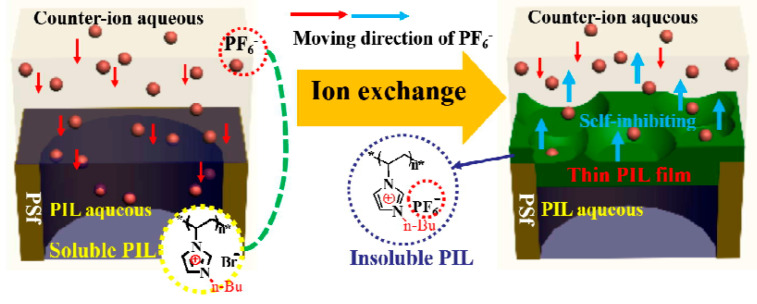
Schematic illustration of the formation mechanism of the poly(ionic liquid) (PIL)/polysulfone (PSf) preparation process. Adapted from [64].

Tang et al. reported a novel method to prepare positively charged NF membrane by using rapid counter-ion exchange of hydrophilic poly(1-vinyl-3-butylimidazolium) bromide (i.e., PIL/polysulfone (PSf) in aqueous KPF6 solution. The system was then transformed from the initial hydrophilic state to a hydrophobic state, and the scheme is shown in Figure 3 [64]. Interestingly, a thin film of hydrophobic PIL layer was formed in the interface of the hydrophilic PIL and KPF6 aqueous phase due to phase separation along with the self-inhibiting effect induced by the hydrophilic–hydrophobic transformation of the PIL chains. The designed PIL/PSf membrane showed pure water permeance (PWP) of 7.55 Lm^−2^h^−1^bar^−1^ (LMH/bar), a rejection of 84% to MgCl_2_, and a high rejection of about 90% to heavy metallic salts.

**Figure 4:**
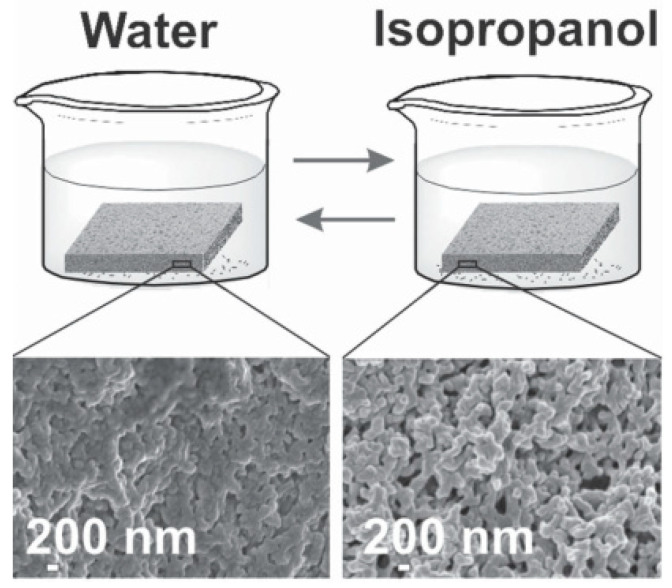
Schematic representation of the solvent experiment by reversibly immersing the membrane in water and isopropanol and the respective SEM images of the membrane structure. Adapted from [65].

Yuan’s group fabricated porous polymeric membranes via simultaneous phase separation of a PIL and its ionic complexation with an acid, which occurred in a basic solution of a nonsolvent [65]. As shown in Figure 4, the membranes have stimuli-responsive porosity. This means they had open pores in isopropanol but close ones in the water, leading to higher isopropanol flux but lower water flux. This property made them potential prospective for stimuli-responsive filtration systems, smart sensors, or controlled loading and release systems. However, further studies are needed to explore their practical applications.

Kohno et al. reported a novel type of thermo-responsive PILM that could control the partition of proteins via a lower critical solution temperature (LCST) behavior for protein concentration from aqueous media [66]. They studied the salt effects on the phase behavior of PIL materials, including PILMs in combination with different IL monomers and salt species. The results showed that the water content of a chemically cross-linked and sufficiently hydrated PILM 1, i.e., poly([P4444] [SS]0.3-co-[P4448] [SS]0.7)-type, exhibited reversible water uptake/release via LCST behavior, enabling selective concentration of proteins without significant loss of their higher-order structures.

**Figure 5:**
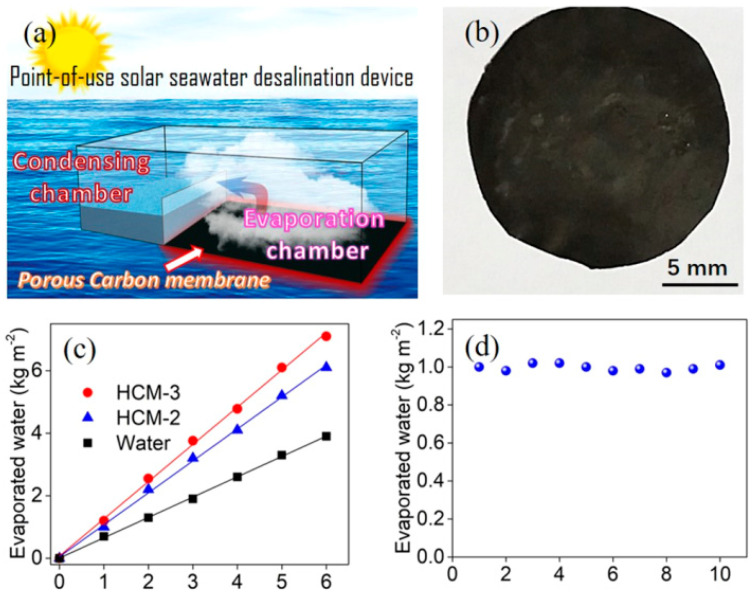
(**a**) Illustration of an air-water interface solar desalination device. (**b**) Digital photographs of hierarchically porous carbon membrane HCM-3 with a diameter of 0.8 cm and thickness of 160 μm. (**c**) Mass of the evaporated water as a function of the radiation time with and without HCMs. (**d**) Reusability of HCM-3 for solar-powered seawater desalination. Adapted from [67].

Besides the direct use of PILs to fabricated liquid-separation membranes, PILs could also be made into porous carbon membranes. For instance, Shao et al. fabricated charged porous membranes (CPMs) with controllable pore architectures by using a rational choice of anions in PILs [67]. Afterwards, they also successfully synthesized hierarchically porous carbon membranes (HCMs) with micrometer-sized pores from CPMs by using one-step vacuum pyrolysis, which was uniform in the molecular distribution of nitrogen species. The HCMs as photothermal membranes exhibited high performance for seawater desalination as shown in Figure 5, revealing their great potential in portable water production technologies. Although there are only a few studies of PILMs for liquid separations at present, they show promising potential. Further investigations could unlock great potential applications and progress in this area.

As indicated by the aforementioned research, PILs are able to form novel structures and morphologies that are not accessible using ILs due to the polymer segments. Accordingly, PILMs have more versatile applications than the IL membranes. Currently, the application of PILMs for liquid separations is still a relatively new research area. The future of this field lies in the development of new polymers and PIL-nanomaterial composites with improved properties.

### 2.2. ILs as Physical Additives

Recall that the various membranes synthesized with ILs as the fabricated materials which we have discussed so far have poor stability in liquid separations due to the loss of the ILs in the liquid phase. As a result, researchers have made efforts to enhance membrane stability and separation performance by utilizing ILs to physically modify polymeric/inorganic membranes. In other words, the ILs were used as physical additives; some representative research using ILs or PILs as such are stated below.

#### 2.2.1. IL-Polymer Blending Membranes for PV

Blending ILs with hydrophobic polymers to fabricate PV membranes for solvent recovery has drawn much attention recently. To fabricate the IL/PIL-polymer membranes, the IL/PIL and polymer are normally mixed and dissolved by solvents to form a polymer solution, which is then cast into a membrane.

For example, Izak’s group impregnated PDMS-1-ethenyl-3-ethyl-imidazolium hexafluorophosphate (IL1) and PDMS-tetrapropylammonium tetracyano-borate (IL2) blend into ceramic ultrafiltration membrane support to fabricate PV membranes for acetone and 1-butanol removal from water [68]. Compared with the pristine PDMS membrane, the PDMS-IL membranes greatly improved the enrichment factor. More importantly, they showed good stability under a low pressure of 20 Pa in an aqueous solution of acetone and 1-butanol for more than five months. In their follow-up work, the diffusion coefficients and sorption isotherms of 1-butanol in the pristine PDMS membrane and two PDMS-IL membranes at different pressures were determined [69]. The results indicated that the higher permeation flux and enrichment factors of the IL-PDMS membranes were probably caused by the higher diffusion coefficient.

**Figure 6:**
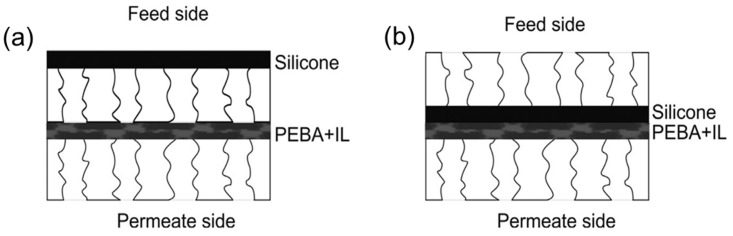
Arrangement of double-layer membranes. (**a**) consists of two separated layers, polyether block amide (PEBA) + IL and silicon layer oriented to the feed side; (**b**) PEBA + IL is located to feed side while the silicon layer is oriented to the permeate side. Adapted from [70].

Rdzanek et al. blended two ILs, namely trihexyl (tetradecyl) phosphonium tetracyanoborate (P_6,6,6,14_ tcb) and 1-hexyl-3-methylimidazolium tetracyanoborate (Im_6,1_ tcb), with polyether block amide (PEBA) and immobilized them into the pores of PSf or polypropylene membranes to fabricate PV membranes for ABE recovery from water [70]. The IL elution into the liquid feed solution could still pose some challenges, since the blending of IL and polymer is based on physical forces. To overcome this issue, the authors covered the PEBA+IL membrane with an additional thin silicone layer using two different arrangements, as shown in Figure 6. The experimental results showed that arrangement (2) was more suitable for the butanol separation because it had a smaller water flux. In their later work, the silicone layer was replaced by PEBA but they still used arrangement (2) and concluded that the addition of a hydrophobic IL in the PV membrane could decrease the water flux and enhance the enrichment factor. However, a long-term PV test of these membranes is still needed to confirm their stability.

Ong and Tan also blended [BMIM][BF_4_] with polyvinylalcohol at a weight ratio of 70/30 and immobilized the [BMIM][BF_4_]- polyvinylalcohol solution into a porous bucky paper made of carbon nanotubes by vacuum filtration to form a PV membrane, which was then cross-linked with glutaraldehyde [71]. The fabricated membrane successfully dehydrated water from a ternary azeotropic mixture of ethyl acetate/ethanol/water. The results suggested that the addition of IL could attain a good balance in the trade-off between the permeation flux and the separation factor.

**Figure 7:**
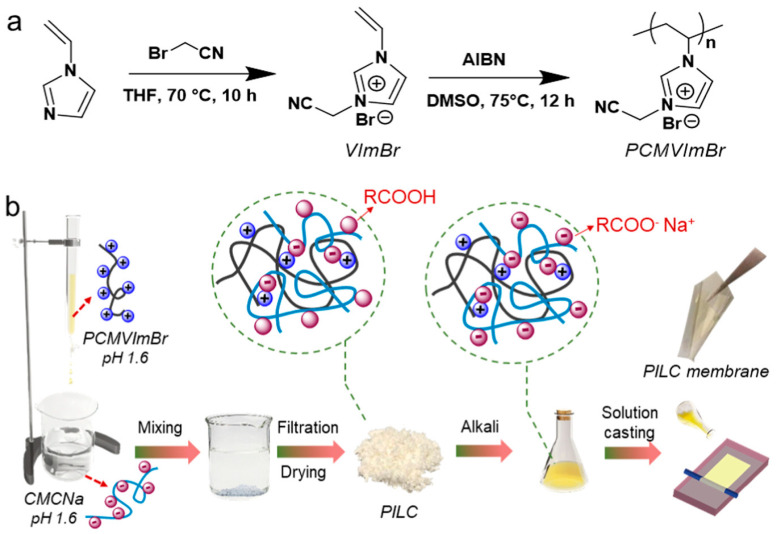
(**a**) Synthesis of IL monomer (VImBr) and its radical polymerization yielding PIL (PCMVImBr), (**b**) schematic preparation of the PCMVIm-CMCNa PILC membranes. Adapted and modified from [72].

Clearly, PILs can also be used as physical membrane additives. Tang et al. synthesized positively charged PIL (poly[1-cyanomethyl-3-vinylimidazolium bromide], PCMVImBr) and blended it with positively charged sodium carboxymethyl cellulose (CMCNa) to form a stable PV membrane based on electrostatic force, and the scheme is illustrated in Figure 7 [72]. The blended membranes with the PIL performed well by stably dehydrating 10 wt% acidic water-isopropanol mixtures. The separation factor was also much higher compared to the pristine CMCNa membrane. 

#### 2.2.2. IL-Polymer Blending Membranes for Separating Metal/Organic from Water

Meanwhile, IL-polymer blending membranes have been used for the separation of organics and various metal ions from aqueous solutions. In this context, He et al. fabricated a cellulose acetate (CA)-sulfonated polysulfone (SPS) blend imprinted membranes for selective adsorption of salicylic acid (SA) by using phase inversion [73]. To enhance the separation efficiency, polyethylene glycol-4000 (PEG 4000) and IL 1-butyl-3-methylimidazolium chloride ([BMIM]Cl) were mixed with the polymer dope as additives. The CA/SPS (90/10) + [BMIM]Cl membranes possessed higher membrane flux, stronger adsorption capacity, and higher selectivity for the SA relative to the competitive species. This performance was attributed to the fact that SPS improved the hydrophilicity of the membrane, whereas the IL promoted the formation of a dense and ordered porous structure. 

Similarly, Chen’s group designed an asymmetric PVDF membrane with addition of IL [tricaprylmethylammonium][di-(2-ethylhexyl)orthophosphinate] ([A336][P507]) for the preconcentration and separation of the heavy rare earth Lutetium [74]. Their study showed that the transport sequence of LuCl_3_ and YbCl_3_ in the membrane was different from that in liquid-liquid extractions, which benefited the separation between Yb and Lu. Moreover, the PVDF-[A336][P507] membrane showed good stability and reusability for LuCl_3_ transport, albeit with weak physical interaction between them.

Elias et al. also fabricated a membrane for mercury preconcentration by incorporating two different ILs: trioctylmethylammonium thiosalicylate (TOMATS) and trioctylmethylammonium salicylate (TOMAS) into cellulose triacetate using nitrophenyl octyl ether as a plasticizer [75]. The membrane with TOMATS yielded effective transport of Hg, which was then made into a special device for global detection of low-concentration Hg in natural water. More interestingly, they investigated the growth of biofilm on the surface of the membrane for the first time and observed no significant differences in Hg transport between a fresh membrane and a membrane deployed for 7 days in a pond. 

**Figure 8:**
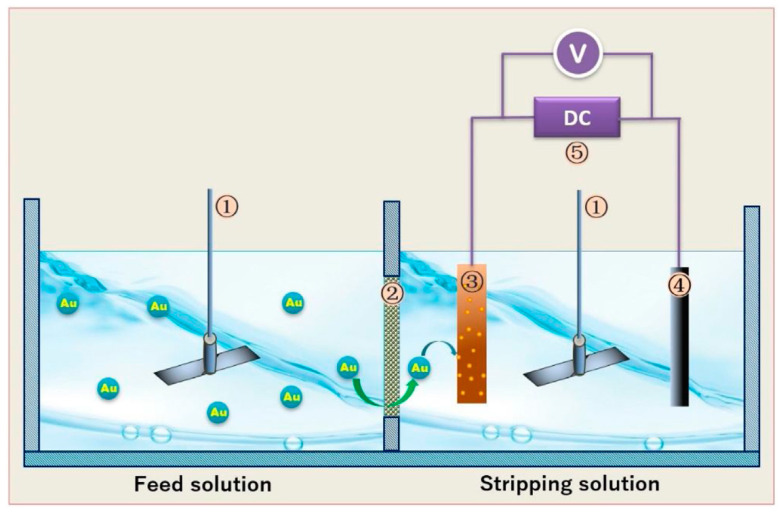
Schematic illustration of the permeation device with an electroplating module. 1: stirrer; 2: PIM; 3: copper cathode; 4: graphite anode; and 5: DC stabilized voltage source. Adapted from [77].

Yang’s group, on the other hand, fabricated a polymer inclusion membrane (PIM) for the separation of low-concentration gold (I) from alkaline cyanide solutions using solvent evaporation with PVDF, [A336][SCN] as the carrier, and 2-nitrophenyl n-octyl ether as the plasticizer [76]. Their results indicated that the IL concentration greatly influenced the extraction because the mechanism involved an anion exchange reaction between the IL embedded in the PVDF and the gold cyanide complex in the feed. The membrane showed a high extraction efficiency of 98.6% for Au(CN)^2−^ and a high gold recovery rate of 98.2% after 24 h using KSCN as the stripping phase. A re-usability investigation confirmed that the PIM maintained long-term stability and excellent durability. In their later work, the PIM system was integrated with an electroplating unit; the scheme is illustrated in Figure 8 [77]. The permeability coefficient of gold increased over two folds after the constant voltage was applied to the stripping solution. Thus, the membrane flux increased with high extraction and deposition percentages of gold. Furthermore, the metallic state gold was coated uniformly on the cathode.

### 2.3. ILs as Chemical Modifiers

In fact, there have been concerns about IL leaching out to the feed phase for the IL-polymer blending membranes, since the interaction between the IL and polymer matrix is based on weak physical forces [70]. Considering that many ILs have functional groups that can covalently be bonded with other materials, researchers have also tried to use IL to chemically modify polymeric/inorganic membrane materials or additives.

#### 2.3.1. Chemically Modify Membrane

On one hand, ILs could chemically bond to polymer chains before forming a membrane, which normally results in a membrane with different membrane structures and possesses improved membrane stability. For instance, Mai et al. fabricated an immobilized [Tf_2_N]^−^ based IL-PDMS membrane to recover acetone, n-butanol, and ethanol (ABE) from an aqueous solution by PV [53]. In order to covalently bond the [Tf_2_N]^−^ based IL to the PDMS backbone polymer, a [Tf_2_N]^−^ based IL precursor which contains active groups which can react with the hydroxyl terminated PDMS was synthesized in advance. Compared with the conventional IL-PDMS supported membrane where the IL was filled in the void volume of the PDMS membrane, the immobilized IL-PDMS membrane exhibited much higher permeate flux, enhanced the recovery of accompanying products such as acetone and ethanol from ABE fermentation, and improved operational stability.

**Figure 9:**
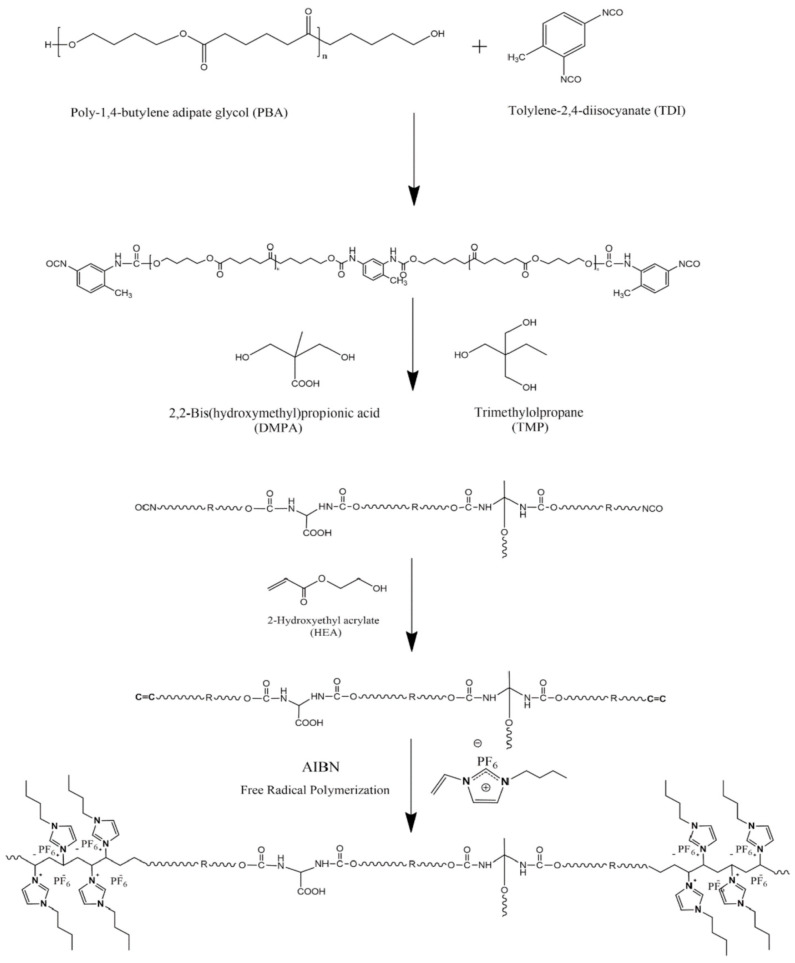
The schematic diagram for the preparation of IL copolymerized waterborne polyurethane (IL-co-PU). Adapted from [78].

Xi et al. synthesized IL copolymerized waterborne polyurethane (IL-co-PU) membranes for the PV separation of benzene/cyclohexane mixtures based on the reaction mechanism shown in Figure 9 [78]. Both the permeation flux and separation factor (benzene/cyclohexane) of the IL-co-PU membranes increased when the IL content was increased, indicating that the IL might facilitate transportation in the membranes.

**Figure 10:**
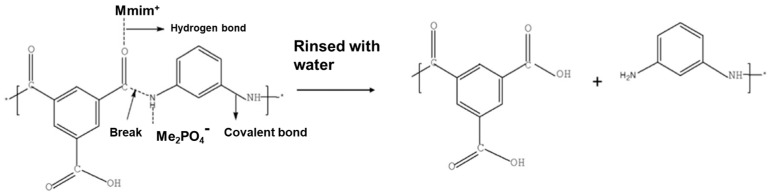
The modification mechanism of the PA active layer in the 1,3-dimethylimidazolium dimethyl phosphate ([MMIM][DMP]). Adapted from [79].

On the other hand, ILs could be chemically bonded to the membrane surface after membrane formation, which tunes the surface properties of membranes and improves the separation performance, antifouling properties, stability, and so on. Most related studies focus on using ILs to surface modify polyamide membranes because the polyamide chains could be split by the hydrogen bonds with imidazolium ILs or react with amine-containing ILs via the “acyl chloride~amine” esterification. Zhang et al. also synthesized an IL (i.e., 1,3-dimethylimidazolium dimethyl phosphate ([MMIM][DMP])), which was adopted to modify the surface of the commercial RO membrane by an activation method [79]. It was revealed (Figure 10) that the IL modification mechanism was based on the effective breakage of inter- and intra-molecular hydrogen bonds in the polyamide chains accompanied by the breakage of polyamide chains dissolved in the IL. The results showed that the modification led to a thinner, smoother, and more hydrophilic PA layer of the RO membrane, resulting in a great improvement in the water permeability and anti-fouling property.

**Figure 11:**
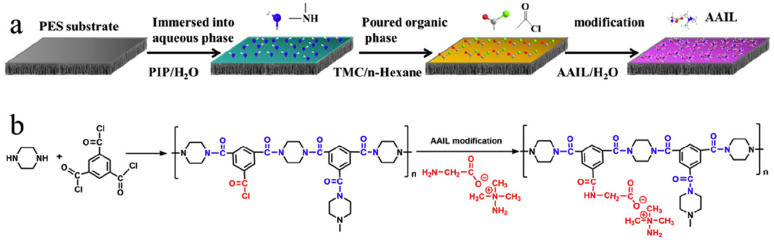
(**a**) Schematic diagram of an amino acid IL (AAIL) modified polyamide selective layer, and (**b**) the possible reaction formula. Adapted from [80].

Sun’s group used an amino acid IL (AAIL) to functionalize interfacial polymerized NF membranes, and the scheme is shown in Figure 11 [80]. The AAIL modification did not only improve the hydrophilicity and increase the pure water permeability by 63%, but it also caused the membrane surface to be more negatively charged, resulting in high Na_2_SO_4_/NaCl selectivity. Furthermore, the amino acid end group of the AAIL could serve as a humectant, allowing the membrane to be heat-treated and stored in a dry state.

**Figure 12:**
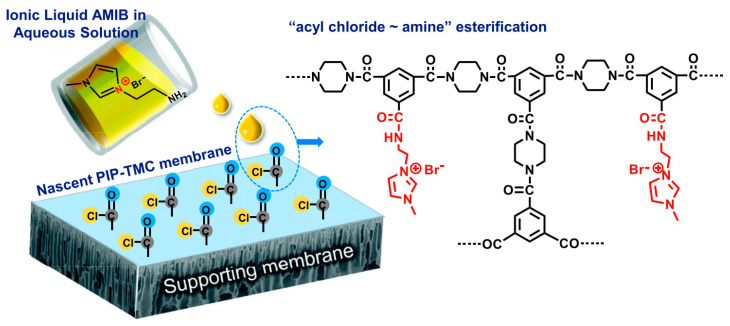
Surface modification of polyamide thin-film composite membranes by 1-aminoethyl-3-methylimidazolium bromide (AMIB). Adapted from [81].

In a similar fashion, He et al. utilized an imidazolium IL (1-aminoethyl-3-methylimidazolium bromide, AMIB) to surface modify the polyamide selective layer of thin-film composite NF membranes via “acyl chloride~amine” amidation, as shown in Figure 12 [81]. They also found a great improvement in the water flux with good salt rejection (R_Na2SO4_ = 95%) after the IL modification. Moreover, the IL modified membrane showed good performance for antibiotic/salt separation as well as promising levels of stability and antibacterial ability.

Liu et al. used IL or polydopamine to chemically modified graphene oxide (that is, iGO or pGO), and then the modified GO nanosheets were assembled with polyelectrolytes on polyethersulfone (PES) membrane support to form composite GO membranes (PE-iGO or PE-pGO) for dye/salt fractionation [82]. The iGO nanosheets were formed by binding methylimidazolium IL with the -COOH groups on GO mediated by 1-ethyl-3-(3-dimethylaminopropyl) carbodiimide hydrochloride and N-hydroxy-succinimide. The PE-iGO membrane could be operated at a low operating pressure of 0.5 bar while achieving high permeability of ~38.4 LMH/bar (100 ppm direct red 80, 5 g/L NaCl). In fact, the salt rejection for 10 g/L NaCl was constantly lower than 5%. Moreover, the IL modification favored the elution of dye molecules from the IL moieties at higher pH, thus improving the efficiency of alkaline washing of the membrane. 

#### 2.3.2. Chemically Modify the Membrane Additives

**Figure 13:**
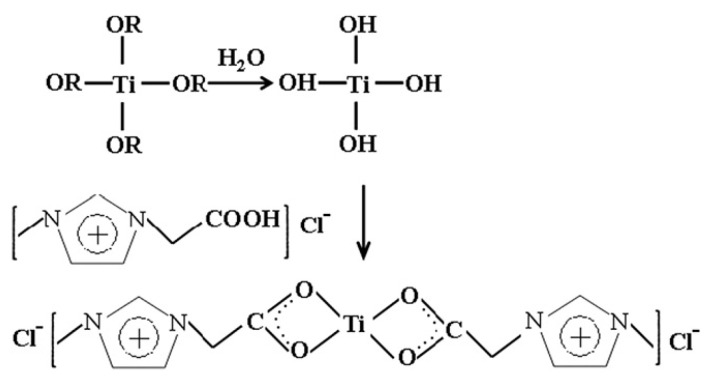
The schematic diagram for the formation of IL-TiO_2_. Adapted from [83].

For these products, Shi et al. synthesized IL-TiO_2_ nanoparticles by chemically modifying TiO_2_ with carboxyl-functional IL ([CH_2_COOHmim]Cl) according to the reaction route shown in Figure 13. The modified product was then added into the PVDF/dimethyl phthalate solutions to fabricate PVDF/IL-TiO_2_ hybrid microfiltration membranes via the thermally induced phase separation (TIPS) method [83]. The addition of IL-TiO_2_ had a strong effect on the crystal formation in the TIPS process. Moreover, the increased amount of IL-TiO_2_ initially increased pure water flux and porosity but then decreased the parameters eventually. However, both the stability and antifouling property were also enhanced, indicating that the PVDF/IL-TiO_2_ hybrid membranes may have potential in catalytic water treatment.

Abraham et al. then fabricated mixed matrix membranes (MMMs) for separating toluene/methanol azeotropic mixtures by incorporating IL (1-benzyl-3-methyl imidazolium chloride) functionalized multi-walled carbon nanotubes into styrene-butadiene rubber (SBR) [84]. The benzyl groups of the IL on the MWCNT surface possessed greater toluene affinity and higher repellency against methanol due to their aromatic π-π interactions with toluene molecules, leading to higher permeation flux and separation factor compared to pristine SBR membranes. 

**Figure 14:**
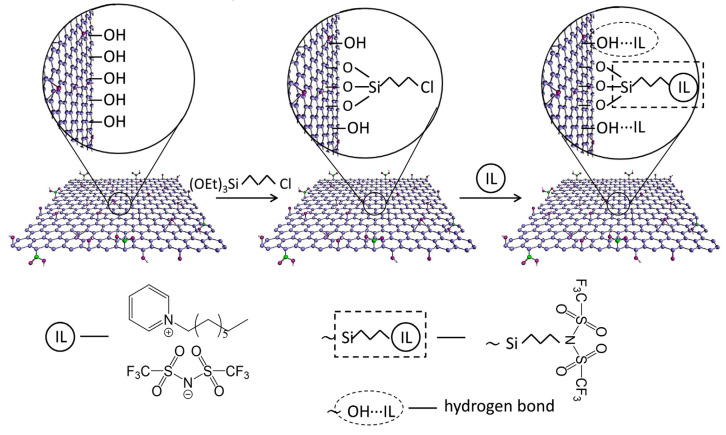
Preparation of IL modified graphene oxide and the structure of N-octylpyridiniunm bis (trifluoromethyl) sulfonyl imide ([OPY][Tf_2_N]). Adapted from [85].

Tang et al. also fabricated pervaporative MMMs for butanol recovery from aqueous solutions by incorporating IL (N-octylpyridiniunm bis (trifluoromethyl) sulfonyl imide, [OPY][Tf_2_N]) modified graphene oxide (IL-GO) nanosheets into PEBA matrix [85]. The reaction mechanism between [OPY][Tf_2_N] and GO is shown in Figure 14. The author also found that the addition of IL-GO increased the separation factor and the permeation flux of the MMMs due to the good butanol affinity as well as the hydrophobicity of the IL. Furthermore, anchoring the IL to GO avoided the IL loss to the feed during the PV process, and thus enhanced the membrane stability. The long-term stability was conducted during a 180 h PV test of the IL-GO/PEBA MMM, in which the separation performance almost showed no change.

**Figure 15:**
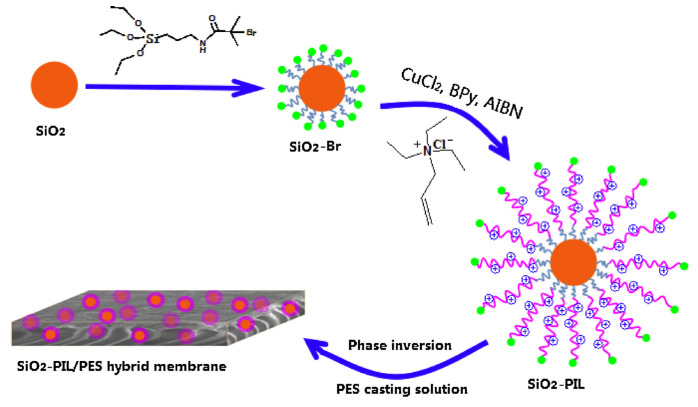
The preparation process for SiO_2_-PIL/polyethersulfone (PES) hybrid membrane. Step 1: the anchoring of the initiator (BTPAm); step 2, the polymerization of IL monomers (ATEA-Cl) on the surface of SiO_2_ particles by using the reverse ATRP method; step 3, the preparation of SiO_2_-PIL/PES positively charged membrane by blending SiO_2_-PIL particles with a PES casting solution. Adapted from [86].

Likewise, PILs have been used for the chemical modifications of membranes. Zhang’s group first synthesized positively charged nano-sized silica spheres modified by PIL brushes via atom transfer radical polymerization (ATRP), and then incorporated them into the PES solution to cast SiO_2_-PIL/PES MMMs for NF (Figure 15) [86]. The designed positively charged MMMs showed higher water flux, low-molecular-weight organic rejection, and salt permeability. Furthermore, the salt concentration showed little effect on the separation property. The authors also used the same PIL to modify Mg-Al hydrotalcite (HT) nanosheets and fabricated HT-PIL/PES MMMs using a similar strategy [87]. The HT-PIL/PES MMM showed higher rejection of reactive dyes than the previously developed SiO_2_-PIL/PES MMMs (90~95% vs 85~95%). This type of loose NF membrane may open opportunities for separating dyes from salts-containing textile wastewater.

### 2.4. ILs as Solvents

ILs have also been used to dissolve polymers; in this case, they serve as green solvents, especially for those polymers which have limited solubility in common organic solvents. This has extended the range of polymers that can be dissolved in order to fabricate membranes for phase separation. Meanwhile, ILs can also serve as reaction media for polymerization or other chemical reactions during membrane fabrication or modification.

#### 2.4.1. Solvents for Polymer Dissolution

The liquid-separation membranes fabricated from the non-solvent induced phase inversion of polymer/ IL solutions, which have been reported in the recent 10 years are summarized in Table 1. 

As shown in Table 1, cellulose is the most widely studied polymer to be dissolved by ILs and cast into liquid-separation membranes by non-solvent phase inversion. This may be because cellulose is the most abundant renewable biopolymer on the earth but it is very difficult to be dissolved and processed using traditional organic solvents. Besides, the most frequently studied ILs for dissolving these polymers are almost all hydrophilic imidazolium cations based ILs. They have a strong ability to disrupt hydrogen bonds in polymers and are also miscible with water to favor the membrane fabrication through non-solvent phase inversion. Related studies in different liquid separation areas are introduced based on the types of polymers used as follows.

Most of the cellulose membranes fabricated from cellulose-IL solutions by water-induced phase inversion have pores that are within the MF or UF range. Some of the MF/UF cellulose membranes have been directly used for the rejection of PEO [89], PEG [94], PS [94], humic acid [90], or oil [91]. There has also been some research work which has succeeded in fabricating cellulose NF membranes. For example, Li et al. fabricated cellulose NF membrane by phase inversion from a cellulose - [AMIM][Cl] solution on PET non-woven fabric [35]. The rejection data for a series of dyes showed that the molecular weight cut-off was less than 700 Da. Falca et al. also fabricated cellulose HF membranes via spinning using three different ILs as solvents [94]. The HFs spun from solutions in [DMIM][DMP] and [EMIM][DEP] showed better results for dye rejection. However, it should be noted that the fabricated membranes showed good rejections for negatively charged dyes but poor rejection for positively charged dyes in water or ethanol, indicating that the charge effect rather than size exclusion was dominant during the separation. Therefore, they may not be suitable for OSN in non-polar solvents. Esfahani et al. fabricated loose NF membranes from cellulose-[BMIM][Cl] solutions; the cellulose was extracted from bamboo waste fiber, indicating the sustainability of the technique [95].

Meanwhile, other membranes fabricated from cellulose-IL solutions were not used directly but have to be further modified before they could be used for liquid separations. For example, Barroso et al. reported on the surface modification of a cellulose MF membrane which could also be surface modified with a synthetic ligand 2-(3-aminophenol)-6-(4-amino-1-naphthol)-4-chloro-s-triazine, and was used to absorb human immunoglobulin G (IgG) rather than BSA, achieving the separation goal [62]. Livazovic et al. designed a polyamide/cellulose TFC membrane by using interfacial polymerization between m-phenylenediamine (MPD) and trimesoyl chloride (TMC) on the cellulose surface. The MWCO was much smaller than f the pristine cellulose membrane ( <200 vs. 3000 Da) [89]. Abdellah et al. fabricated a polyester/cellulose TFC membrane by using the interfacial reaction between catechin and terephthaloyl chloride [92]. The membrane showed dimethylformamide permeance of 1.2 LMH/bar with an MWCO around 500 Da. The membrane exhibited stable performance within 1 month, indicating great potential for application in the food and pharmaceutical industries.

Besides, other materials could be added to the cellulose-IL solution to fabricate the relevant cellulose composite membranes. In this case, Nevstrueva et al. reported that adding a small amount of TiO_2_ can increase the PWF of the resultant membrane but also that doing this reduced the humic acid retention [90]. Colburn et al. incorporated graphene oxide quantum dots (GQDs) into cellulose. The abundant hydroxyl and carboxyl groups of the GQD made the cellulose membrane very stable due to the hydrogen bonding, made it negatively charge, and made it more hydrophilic. In their later work, iron oxide nanoparticles, polyacrylic acid, and lignin sulfonate were uniformly incorporated into their cellulose membranes [93]. The iron-cellulose membrane showed excellent dye rejections with MWCO less than 300 Da. Slusarczyk and Fryczkowska also reported that the introduction of nano-sized GO into the cellulose matrix influenced the membrane formation process, consequently, the physicochemical, transport, and separation properties of the composite membranes [36]. Yet, the addition of GO enhanced the PWF by up to 10 fold, with a rejection of heavy metals reaching higher than 90%.

The applications of ILs become very meaningful when they are used to dissolve polymers (e.g., cellulose, PBI, PMIA, polytriazole, etc.) which are not easily dissolved by traditional organic solvents. Chung’s group used [EMIM][OAc] to dissolve the rigid polymers PBI [26] and PMIA [27], and fabricated membranes for OSN. The chemically crosslinked PBI membrane displayed good separation performance and impressive stability in many aggressive solvents. While the glutaraldehyde (GA) modified PMIA membranes with a selective layer synthesized by the water-based reaction between GA and hyper-branched polyethylenimine (HPEI) showed MWCO of 470-730 Da with acceptable ethanol permeance. Importantly, they also showed good performance for concentrating lecithin in hexane. Nunes’s group utilized [EMIM][DEP] to dissolve polytriazole, followed by nonsolvent induced phase inversion and chemical crosslinking to fabricate effective membranes [101]. The crosslinked polytriazole membranes showed good performance for dipolar aprotic solvents (e.g., N, N’-dimethylformamide) with a MWCO within 1~3 kDa with an operational temperature varying from 25 ^o^C to 105 ^o^C, which have great potential for applications under high-temperature harsh organic solvent environments.

The ILs have also been studied to dissolve other polymers which are easier to dissolve by using traditional organic solvents. This is because the nonvolatility of ILs avoids the emission of volatile organic compounds that are toxic. Cellulose acetate, a derivative polymer of cellulose, was also dissolved in imidazolium cations based ILs to fabricate flat sheet [96,97] or HF [24] membranes with a pore size in the UF range. However, these membranes were only suitable for the separation of aqueous solutions because cellulose acetate is not as stable as cellulose in organic solvents. Besides, other polymers such as Extem, PVDF-HFP, and P84/PBI blend were also dissolved by using ILs and they can be fabricated into UF or MF membranes [98,99,100]. In addition, researchers are using IL-organic solvent mixtures to dissolve polymers in order to reduce the viscosity of the polymer solution for easier processing and/or adjusting the membrane structure [102,103,104]. In these cases, the selection of less toxic organic solvents is critical due to environmental factors.

Overall, ILs are good solvents for dissolving polymers for fabricating membranes. They have a strong ability to dissolve rigid polymers. More importantly, the negligible vapor pressure of ILs makes the membrane fabrication processes more environmentally benign. However, the types of ILs that could be used for the dissolution of polymers are limited currently. Moreover, the mechanism for the dissolution of polymers in ILs and the phase inversion of IL-polymer solutions to form membranes are not widely studied, making the selection of suitable ILs for desired polymers time-consuming.

#### 2.4.2. Solvents as Reaction Media during Membrane Fabrication

ILs could also serve as solvents (reaction media) for the interfacial polymerization reaction for fabricating TFC membranes. For instance, Vankelecom’s group conducted several studies on this research area in recent years. A water-immiscible IL, [BMIM][Tf_2_N] was used as the organic reaction phase for the interfacial polymerization between MPD and TMC to form the TFC RO membrane. The membrane showed 350% higher water permeance with comparable selectivity (96.8% NaCl retention) to the TFC membranes synthesized using traditional hexane as the organic phase [28]. In their subsequent work, the synthesis conditions were further optimized in terms of reaction time, cost-efficiency, and environmental impact [105]. The time for interfacial polymerization was reduced to 10 seconds. More importantly, the IL and TMC could be recycled, proving the eco-friendliness and sustainability of their technique. In the meantime, a polyamide/crosslinked Matrimid TFC membrane for FO was fabricated by using [BMIM][Tf_2_N] or [BMIM][Tf_2_N]/hexyl acetate mixture as the organic phase for the interfacial polymerization [106]. The use of the IL instead of hexane made the fabrication process less hazardous but with similar FO separation performance. These studies have shown the great potential of ILs as the reaction media for interfacial polymerization in fabricating liquid-separation TFC membranes. However, the ILs which could be used for such purposes are still very limited, and the reaction mechanism and reaction kinetics need to be studied further to precisely control such reaction processes.

## 3. The Sustainability of ILs in Developing Liquid Separation Membranes

More and more ILs have been explored in assisting the development of liquid separation membranes. The information of the commercial ILs used in membrane fabrication is summarized in Table 2. As shown, the prices of most ILs are much higher than common organic solvents. Besides this economic aspect, the environmental aspects of ILs such as stability, biodegradability, toxicity should be considered as well [107,108,109]. These aspects are major hurdles for sustainable development and commercialization of the ILs related technologies. Therefore, the recovery of ILs is of great importance. Various separation methods have been developed for the regeneration, recovery, or removal of ILs, including phase separation methods (i.e., evaporation, vacuum distillation, and crystallization), phase addition methods (i.e., liquid-liquid extraction and supercritical fluid extraction), adsorption by solid agents or solid extraction, separation by a barrier (i.e., membrane filtration methods and PV), separation by an external force-field (e.g., decantation, magnetic-field separation), which have been comprehensively summarized by several review papers [108,110,111,112]. 

Even though versatile methods for recovering ILs have been studied, suitable ones should be chosen by analyzing the physicochemical properties of ILs and other components. Among cases of using ILs for fabricating liquid separation membranes, the ILs recovery is most urgent in the case where ILs are used as solvents to dissolve the polymers or as the reaction media during membrane fabrication because a large amount of ILs is used.

In the former situation, the mixture to be separated is normally composed of ILs and water. Xing et al. used a simple evaporation method to remove the water in a coagulant bath, and thus recovered [BMIM][SCN] after the membrane fabrication [96]. Interestingly, the recovered [BMIM][SCN] was reused for casting cellulose acetate flat sheet membranes, which showed morphological and performance characteristics similar to those prepared by using fresh [BMIM][SCN]. However, the coagulant bath contains much water, thus making the evaporation method energy-consuming. Therefore, more research focuses on using NF for recovering ILs from aqueous solutions [110,112]. In the latter situation, the recovery of ILs is a bit difficult because the ILs contain monomers (precursors of the thin-film composites) that are also not volatile. As aforementioned, Vankelecom’s group fabricated TFC RO membrane via the interfacial polymerization between MPD and TMC using [BMIM][Tf_2_N] as the organic reaction phase. Though TMC and [BMIM][Tf_2_N] were not separated, the mixture could be recycled several times for membrane synthesis, without loss of performance [105].

Nevertheless, the studies on the regeneration, recovery, and removal of ILs after the fabrication of liquid-separation membranes are limited. Future work should provide more efforts towards comparing these methods and finding suitable and efficient methods for different special cases in developing liquid-separation membranes, which would boost the usage of ILs to be more sustainable and beneficial. 

## 4. Conclusions and Future Directions

There have been increasing studies on the use of ILs to fabricate liquid-separation membranes in the last decade. Therefore, four major functions of ILs in developing these membranes have been highlighted and discussed. Consequently, some conclusions and future directions are listed as follows:

(1) Membranes that are fabricated directly with ILs (i.e., BILM, EILM, and SILM) are mainly used for removing organics and metal ions based on extraction. Such membranes often suffer from poor stability due to either the leaching of ILs into the liquid phase or the emulsion swelling and breakage (specifically involving EILM).
(2) PILMs are more stable than IL membranes due to their larger molecular structures. They exhibited good performance in applications such as the removal of metal ions and organic dyes, desalination, the concentration of proteins, and oil/water separation. The PILMs have shown great potential but further studies on them are required.
(3) The stability of the membranes can be improved tremendously if ILs/PILs are blended with polymers due to physical interactions such as hydrogen bonds, π-π stacking, or electrostatic interactions. These types of membranes have been widely studied for organophilic PV and separation of metal/organic from water. However, the issue of IL leaching may still exist due to weak physical interactions.
(4) ILs/PILs can be used to chemically modify polymeric membranes or membrane components (like fillers) to improve the separation performance and membrane stability due to their strong covalent bonds. These membranes showed promising performance and excellent stability in PV, RO, MF, and RO, etc. Some large-scale demonstrations are needed to promote industrial applications.
(5) The use of ILs as a solvent to dissolve polymers (especially those which are too rigid to be dissolved by traditional solvents) and as reaction media for the interfacial polymerization to fabricate liquid membranes are important because this option is less hazardous and more sustainable. Currently, the use of ILs to dissolve polymers and fabricate membranes have been studied a great deal. Therefore, further efforts must be directed to the study of the mechanisms for the dissolution and phase inversion of IL-polymer solutions. Since the use of ILs as reaction media for the thin-film membranes just started recently, more ILs must be investigated for applications in this area, and their reaction mechanism must be explored to fully understand how to precisely control their reaction processes.
(6) To make the usage of ILs in developing liquid separation membranes more sustainable and economic, more efforts should be paid to looking for efficient methods for the regeneration, recovery, and removal of ILs in these special cases.

## Figures and Tables

**Table 1:** Membranes fabricated from polymer/ IL solutions and their applications.

Polymer	IL	Applications	Year	Reference
Cellulose (surface modified)	[BMIM][Cl]	Human immunoglobulin G (IgG) purification by absorption	2010	[88]
Cellulose	[AMIM][Cl]	NF: dye rejection (<700 Da)	2011	[35]
Cellulose (TFC)	[EMIM][OAc]	UF: PEO rejection (3000 Da) NF: PEG rejection (<200 Da)	2015	[89]
Cellulose -TiO_2_	[EMIM][OAc]	UF: humic acid (100 kDa) rejection	2015	[90]
Cellulose	[EMIM][OAc]	UF: oil/water separation	2018	[91]
Cellulose TFC	[EMIM][OAc]	OSN: dye rejection (500 Da)	2018	[92]
Cellulose-graphene quantum dot	[EMIM][OAc]	NF: dyes (300 < MWCO < 5000 Da) rejection	2018	[34]
Cellulose-iron/polyacrylic acid/lignin sulfonate	[EMIM][OAc]	NF: dye rejection (<300 Da)	2019	[93]
Cellulose HF	[EMIM][OAc] [EMIM][DEP] [DMIM][DMP]	UF: PEG rejection (~18 kDa) PS rejection (25 kDa) NF/OSN: Dye rejection (700–1500 Da)	2019	[94]
Cellulose-GO	[EMIM][OAc]	NF: heavy metal removal	2019	[36]
Cellulose from bamboo	[BMIM][Cl]	NF: dye rejections	2020	[95]
Cellulose acetate	[BMIM][SCN]	UF: PEG/PEO rejection	2010	[96]
Cellulose acetate HF	[BMIM][SCN]	UF: PEG/PEO rejection	2011	[24]
Cellulose acetate	[EMIM][OAc]	UF: BSA (66 kDa), γ-globulin (~140 kDa) rejection	2016	[97]
PBI	[EMIM][OAc]	OSN: dye rejection (600 Da)	2014	[26]
PBI/P84	[EMIM][OAc]	UF: PEG/PEO rejection (~100 kDa)	2013	[98]
Extem	[EMIM][SCN]	UF: BSA (66 kDa), γ-globulin (~140 kDa) rejection DNA (6.4 kDa)	2017	[99]
PVDF-HFP	[dema][TfO] [MIM] [Tf_2_N] [MIM][Cl]	MF	2018	[100]
PMIA-TFC	[EMIM][OAc]	OSN: Dye rejection (470–730 Da)	2018	[27]
Polytriazole	[EMIM][DEP]	OSN: PEG rejection rom DMF (1~3 kDa)	2020	[101]

Note: PBI: polybenzimidazole; PVDF-HFP: poly(vinylidene fluoride-co-hexafluoropropylene); PMIA: Poly(m‑phenylene isophthalamide); TFC: thin-film composite; PEO: poly(ethylene oxide), PEG: poly(ethylene glycol); [BMIM][Cl]: 1-butyl-3-methylimidazolium chloride, [BMIM][SCN]: 1-butyl-3-methylimidazolium thiocyanate; [AMIM][Cl]: 1-allyl-3-methylimidazolium chloride; [EMIM][OAc]: 1-ethyl-3-methylimidazolium acetate; [EMIM][DEP]: 1-ethyl-3-methyimidazolium diethyl phosphate; [DMIM][DEP]:1,3-dimethylimidazolium dimethyl phosphate; [dema][TfO]: trifluoromethanesulfonate; [MIM][Tf_2_N]: 1-methylimidazolium bis(trifluoromethylsulfonyl); [EMIM][Cl]: 1-ethyl-3-methylimidazolium chloride; [MIM][Cl]: 1-methylimidazolium chloride; [DMIM][DMP]: 1,3-dimethylimidazolium dimethyl phosphate; BSA: Bovine serum albumin.

**Table 2:** The information of the commercial ILs used in membrane fabrication.

Chemical Name	Abbreviation	Formula	CAS registry Number	Molecular Weight	Density ^a^ (kg/m^3^)	Viscosity ^a^ (Pa×s)	Price (RMB, 100 g Weight Basis) ^b^
1-butyl-3-methylimidazolium tetrafluoroborate	[BMIM][BF_4_]	C_8_H_15_BF_4_N_2_	174501-65-6	226.03	1201	0.108	400
1-butyl-3-methylimidazolium bis(trifluoromethylsulfonyl)imide	[BMIM][Tf_2_N]	C_10_H_15_F_6_N_3_O_4_S_2_	174899-83-3	419.36	1436	0.0508	1500
1-octyl-3-methylimidazolium chloride	[OMIM][Cl]	C_12_H_23_ClN_2_	64697-40-1	230.78	1013	13.3	300
1-butyl-3-methylimidazolium hexafluorophosphate	[BMIM][PF_6_]	C_8_H_15_F_6_N_2_P	174501-64-5	284.19	1367	0.274	400
1-octyl-3-methylimidazolium hexafluorophosphate	[OMIM][PF_6_]	C_12_H_23_F_6_N_2_P	304680-36-2	340.29	1236	0.691	500
1-octyl-3-methylimidazolium bis(trifluoromethylsulfonyl)imide	[OMIM][Tf_2_N]	C_14_H_23_F_6_N_3_O_4_S_2_	862731-66-6	475.47	1320	0.0931	1500
N-butylpyridinium tetrafluoroborate	[BPy][BF_4_]	C_9_H_14_BF_4_N	203389-28-0	223.02	1214	0.1603	500
1-butyl-3-methylimidazolium chloride	[BMIM][Cl]	C_8_H_15_ClN_2_	79917-90-1	174.67	1082	0.00604	200
N-octylpyridiniunm bis (trifluoromethyl) sulfonyl imide	[OPY][Tf_2_N]	C_15_H_22_F_6_N_2_O_4_S_2_	384347-06-2	472.47	1327	0.1143	1600
1-allyl-3-methylimidazolium chloride	[AMIM][Cl]	C_7_H_11_ClN_2_	65039-10-3	158.63	1166	0.82	300
1-ethyl-3-methylimidazolium acetate	[EMIM][OAc]	C_8_H_14_N_2_O_2_	143314-17-4	170.21	1100	0.1436	900
1-ethyl-3-methyimidazolium diethyl phosphate	[EMIM][DEP]	C_10_H_21_N_2_O_4_P	848641-69-0	264.26	1144	0.41	400
1-butyl-3-methylimidazolium thiocyanate	[BMIM][SCN]	C_9_H_15_N_3_S	344790-87-0	197.30	1070	0.05652	1600

^a^ Data was measured at a temperature of 298.15 K and pressure of 100 kPa. Database: https://ilthermo.boulder.nist.gov/. ^b^ Data was provided by Lanzhou Greenchem ILs, CAS, China.
